# Single Trocar Transanal Endoscopy in a Child

**Published:** 2013-05-30

**Authors:** Ciro Esposito, Maria Escolino, Giuseppe Ascione, Alessandro Settimi

**Affiliations:** Department of Pediatrics and Pediatric Surgery, Federico II University of Naples, Via Pansini 5 80131, Naples Italy

**Keywords:** Single trocar transanal endoscopy, Rectal lesion, Transanal surgery, Child

## Abstract

Single trocar transanal endoscopic surgery (STTE) is a well-known technique for evaluation and management of rectal lesions in adults. We used an 11mm balloon trocar with a 10mm operative optic, introduced into the anal canal to excise a rectal lesion along with multiple rectal biopsies in a child. Rectal visibility was good with an ease to maintain the rectum insufflated.

## INTRODUCTION

STTE is a minimally invasive procedure for the removal of rectal lesions, an approach first proposed by Buess et al in 1985.[1] Compared with traditional anal excision or colonoscopy, the advantages of STTE include an excellent visibility of the rectum, increased possibility of obtaining clear margins, and opportunity of approaching and resecting higher rectal lesions.[2] In pediatric patients, STTE was not practiced. We report the details of the first patient treated in our unit using this procedure.

## CASE REPORT

An 8-year-old boy with severe iron deficiency anemia (Hemoglobin 4.7 g/dl), rectal bleeding, and constipation underwent colonoscopy, which showed severe colitis and an intensely hyperemic and friable mucosa with multiple erosions on the postero-lateral wall of the rectum located 4cm proximal to the anal verge. Colonoscopic biopsies showed hyperplastic granulation tissue. For the persistence of the symptoms, repeat colonoscopy with deeper biopsies were performed which revealed polypoid ulcers with cobblestone appearance. Histopathology again showed ulcerated polypoid granulation tissue with reactive epithelial changes. For the persistent constipation and abdominal distension, he underwent a barium enema that showed a dilatation of the rectosigmoid area with fecal loading till descending colon. An entero-MRI was performed which showed marked and diffuse thickening (maximum thickness 8 mm) of the walls of the rectum and distal sigmoid colon with the stenosis of the lumen. The nature of the lesions was still unclear; therefore, an excisional biopsy was performed using STTE with an 11mm balloon trocar (Covidien). In addition, for the persistence of constipation, some biopsies on the antimesenteric margin of the upper part of rectum were performed with laparoscopy using 3 5-mm ports in order to exclude other pathologies.

As for STTE, the anus was gently dilated, and a lubricated 11mm balloon trocar was introduced into the anal canal (Fig. 1, 2). Insufflation was maintained to 15mmHg through the insufflation-access port using CO2. The rectum was sufficiently insufflated to allow a good exposure. There was no leakage of insufflated CO2. A 10mm operative scope with a 5mm operative channel was introduced through the uppermost cannula. The lesion was localized, excised using scissors and monopolar coagulation (Fig. 3). In addition multiple rectal biopsies were performed 2 to 4 cm above the anal verge using a bioptic 5mm forceps. Histopathology showed only inflamed and ulcerated granulation-type tissue and the typical characteristics of a chronic inflammatory bowel disease, more likely a Crohn's disease. Patient was discharged the day following procedure. No adverse events were noted to date, including fecal incontinence. He was treated with medical therapy with good control of the symptoms.

**Figure:**
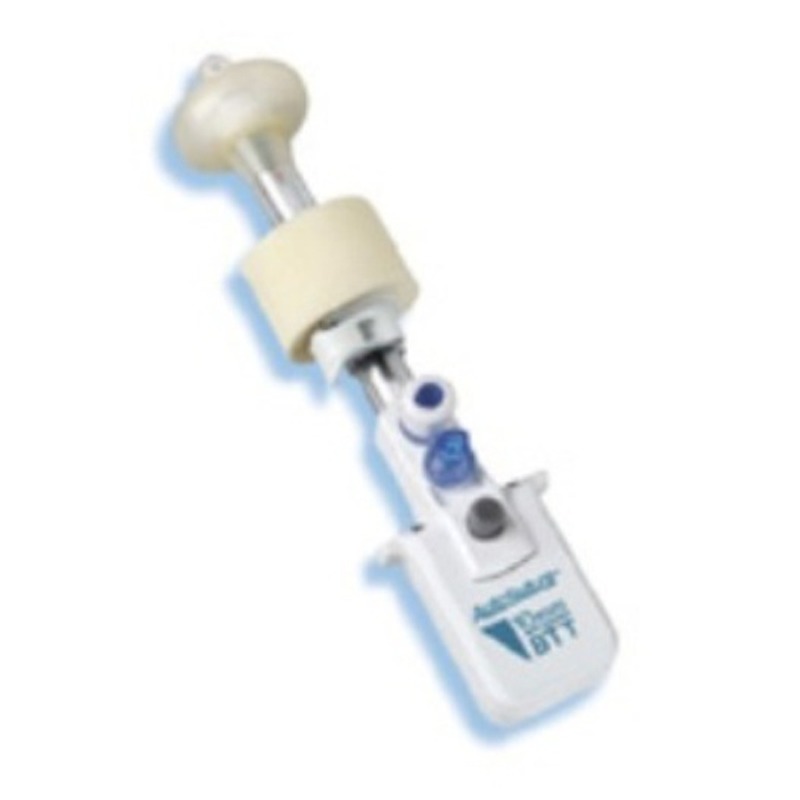
Figure 1: Balloon trocar

**Figure:**
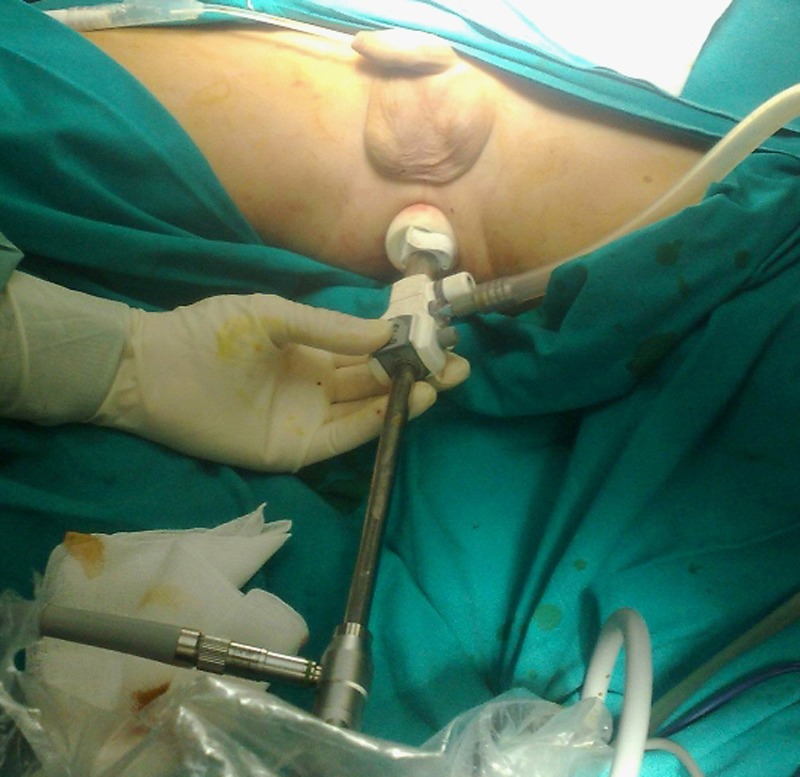
Figure 2: Trocar positioning for the procedure

**Figure:**
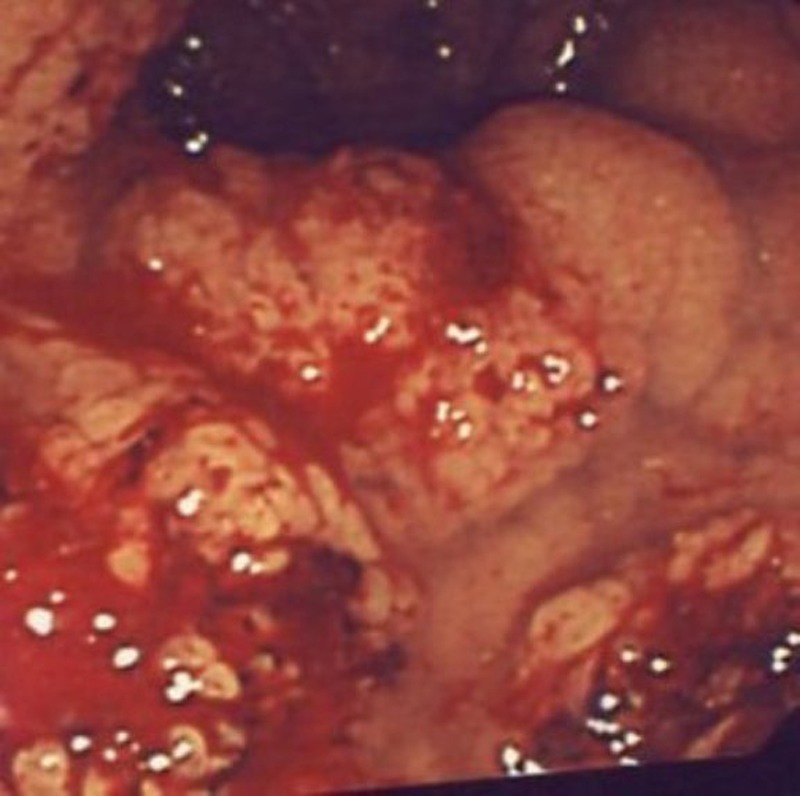
Figure 3: Showing good visibility

## DISCUSSION

STTE has been shown to be effective for the local excision of benign and stage 1 malignant neoplasm in adults [3, 4]. From 2010 few case reports describing a new technique for local excision of rectal tumors using a single-access laparoscopic port have appeared [5]. Recently, van den Boezem et al reported their experience with transanal single-port surgery for the excision of large polyps in adult patients [6]. No report of STTE utilization in pediatric age group is available so far. 

There were no gas leaks during rectal insufflations due to the distal balloon and the sponge around the cannula of the trocar. STTE, with a single port, revealed a practical alternative, particularly for surgeons who have laparoscopic skills. No additional equipment is necessary because standard laparoscopic instruments can be used. Throughout the procedure, the visibility in the rectum was excellent because of the ability to maneuver the camera from side to side, as well as to move it forward, allowing visualization of the upper border of the lesions. This procedure was performed in collaboration with gastroenterologists who found that STTE gives a better, larger and wider view compared to colonoscopy and above all 5mm instruments may be used; so it is possible to perform an endorectal resection much better than with colonoscopy. The benefits found suggest that STTE with a single port may be used for suitable cases. As for technical point of view, an insufflation pressure of 15mm of Hg was used without problem, but probably the pressure should be kept low at about 10mm of Hg. 

We think that it can be an alternative to colonoscopic procedures for example to resect large rectal polyps difficult to remove using standard endoscopic procedures. The procedure seems promising; however, further experience is still necessary to establish the meaningful superiority of this technique over colonoscopy and traditional transanal surgery.

## Footnotes

**Source of Support:** Nil

**Conflict of Interest:** None declared

